# Markers of Maternal Insulin Resistance and Lipid Ratios Measured in Early Pregnancy Are Related to Adverse Fetomaternal Outcomes in Women Treated for Hyperglycemia Detected in Early Pregnancy—Data from a Retrospective Cohort Study

**DOI:** 10.3390/jcm11071777

**Published:** 2022-03-23

**Authors:** Agnieszka Zawiejska, Katarzyna Wróblewska-Seniuk, Paweł Gutaj, Joanna Kippen, Anna Gomulska, Ewa Wender-Ozegowska

**Affiliations:** 1 Department of Medical Simulation, Chair of Medical Education, University of Medical Sciences, 61-701 Poznan, Poland; 2 Department of Newborns’ Infectious Diseases, Chair of Neonatology, Poznan University of Medical Sciences, 61-701 Poznan, Poland; kwroblewska@ump.edu.pl; 3 Department of Reproduction, Poznan University of Medical Sciences, 61-701 Poznan, Poland; pgutaj@o2.pl (P.G.); ewozegow@ump.edu.pl (E.W.-O.); 4 Students’ Scientific Society, Poznan University of Medical Sciences, 61-701 Poznan, Poland; joannakippen@gmail.com (J.K.); klinrozrod.gpsk.um@gmail.com (A.G.)

**Keywords:** gestational diabetes mellitus, diabetes diagnosed in pregnancy, insulin resistance, lipid ratios, metabolic syndrome, fetal growth, early life origins of health and disease

## Abstract

Background: Hyperglycemia detected in early pregnancy is still inadequately studied as a risk factor for adverse maternal and neonatal outcomes. Methods: a retrospective study of a cohort of N = 193 women in singleton pregnancies with hyperglycemia diagnosed before the 20th gestational week (GW). Results: characteristics of the study group: GW at the diagnosis: 12.0 (9.0; 15.0), diabetes diagnosed in early pregnancy (eDiP): 21%, insulin-therapy required: 61.8%, gestational hypertension/preeclampsia: 7.7%, premature delivery: 9.2%, composite adverse neonatal outcome: 59.2%, high (LGA) birth weight/low (SGA) birth weight according to the WHO growth charts: 24.2%/9.2%, respectively. Women with eDiP have lower eGDR, a higher TAG/HDL ratio, and a higher atherogenic index of plasma (AIP) compared to women with gestational diabetes diagnosed in early pregnancy—eGDM (9.33 ± 1.56 vs. 7.92 ± 2.54, *p* = 0.007, 1.06 ± 0.78, vs. 1.25 ± 0.68, *p* = 0.020, and −0.06 ± 0.25 vs. 0.04 ± 0.23 *p* = 0.021, respectively). NonHDL/HDL cholesterol ratio > 2.6, and AIP > 0.24 total/HDL cholesterol ratio > 4.5 significantly predicted metabolic adverse neonatal outcome (hypoglycemia and/or hyperbilirubinemia)—OR (95% CI): 4.62 (1.35; 15.79), 3.60 (1.04; 12.48), 8.75 (1.02; 74.83), respectively. Conclusions: 1, Hyperglycemia diagnosed in early pregnancy coexists with a lipid profile suggestive of insulin resistance. 2, Lipid-related markers of cardiometabolic risk measured in early pregnancy can be useful tools in assessment of fetomaternal risk in high-risk populations. 3, Women with eDiP present a more severe insulin resistance phenotype than those with eGDM.

## 1. Introduction

Gestational hyperglycemia (diagnosed as two, mutually exclusive, entities: gestational diabetes (GDM) or diabetes diagnosed in pregnancy (DiP)) complicates an increasing number of pregnancies, and is currently recognized as one of the leading reasons for maternal and neonatal morbidity worldwide [1]. A typical disease model requires hyperglycemia to be diagnosed between 24–26 weeks of gestation. Therefore, most data regarding fetomaternal complications come from populations with hyperglycemia diagnosed in the third trimester. However, as glucose checks in early pregnancy become part of routine antenatal care, we gather an increasing amount of clinical data that link biomarkers of glucose metabolism from the early gestational weeks to the perinatal outcomes.

There is still some controversy on how to interpret glycemic levels measured in early pregnancy within routine antenatal care, which diagnostic thresholds should be used at this stage of pregnancy, and whether hyperglycemia diagnosed in early pregnancy constitutes a pregnancy-related entity or should be taken as a symptom of pregestational glucose intolerance, diagnosed during pregnancy. Moreover, there remains some uncertainty about the clinical usefulness of early diagnosis and treatment of gestational hyperglycemia, as studies evaluating perinatal outcomes in women treated for hyperglycemia in early pregnancy present conflicting results [2,3].

Fasting hyperglycemia is among the symptoms of metabolic syndrome. Therefore, hyperglycemia diagnosed in early pregnancy, particularly as fasting hyperglycemia in women with overweight or obesity, raises suspicion of combined metabolic disturbances present prior to the pregnancy.

Metabolic syndrome is commonly recognized as a critical measure of cardiometabolic risk, and a major risk factor for type 2 diabetes mellitus [4]. Recent large-scale studies widely report an increasing proportion of the pregnant population with MS present before pregnancy, and call for a model of continuous care tailored to address specific needs of the female population [5,6].

Furthermore, although maternal hyperglycemia is a well-known risk factor for adverse neonatal outcomes, there is an increasing amount of evidence to suggest other non-glycemic risk factors for perinatal complications [7,8]. Maternal obesity is acknowledged as an independent contributor to the complications commonly seen in modern fetomaternal care. [9,10,11]. Recent studies also present strong evidence for an association between maternal lipids and excessive fetal growth or increased neonatal adiposity in the general pregnant population [12,13,14,15]. However, little is known about the gestational lipid profile in the population at an elevated risk of hyperglycemia diagnosed in pregnancy [16,17]. Moreover, there are hardly any attempts to gain an insight into lipid ratios in pregnancy, which could provide new evidence on fetomaternal health in the metabolically high-risk population [18,19]. Data from research regarding the cardiometabolic profile in nonpregnant women show that these lipid markers provide even more reliable insight into the cardiovascular risk in the female population [20,21,22].

In Poland, the IADPSG diagnostic criteria are used to diagnose hyperglycemia (GDM or DiP) throughout pregnancy, both for general testing between 24–26 weeks of gestation, and for testing the high-risk population in the early pregnancy [23]. Women with hyperglycemia diagnosed before the gestational age recommended for a routine diagnostic procedure should be offered antenatal care in referral units dealing with pregnancies complicated by pregestational diabetes. Therefore, we collected observational data related to maternal and neonatal outcomes in a group of women tested for, and diagnosed with, hyperglycemia in the first half of pregnancy, defined as early pregnancy.

We hypothesized that hyperglycemia detected in early pregnancy (i.e., eGDM or eDiP) is associated with an unfavorable lipid profile, expressed as atherogenic lipid ratios, associated with adverse maternal and neonatal outcomes. Our second hypothesis was that hyperglycemia detected in early pregnancy is associated with other surrogate biomarkers of insulin resistance in the first trimester of pregnancy.

## 2. Materials and Methods

To answer our research question, we designed an observational study. We reviewed data of patients referred to the tertiary Unit (University Hospital, Poznan University of Medical Sciences) due to hyperglycemia diagnosed before the 20th week of gestation (eGDM or eDiP) between 2007–2017. We considered all these women as eligible for the study. Next, we excluded women in multiple pregnancies, with miscarriages, late fetal deaths, lacking late pregnancy data, or diagnosed after the 20th week of gestation. Finally, we enrolled the data of 193 women with hyperglycemia diagnosed in early pregnancy to the part of this study referring to maternal outcomes, and 164 mother–infant pairs to the arm of the study investigating neonatal outcomes.

The Bioethics Committee at Poznan University of Medical Sciences reviewed the protocol, and confirmed that our research was not a medical experiment. Therefore, the protocol was exempted from the Committee’s approval (decision No 1321/18). All biochemical tests were performed as a part of routine antenatal care in the accredited laboratory of the University hospital, which holds certificates of quality management ISO 9000.

Upon the first admission, all referred women participated in dietary treatment and glucose self-control training. In women with eDiP, insulin therapy in a basal-bolus mode was initiated immediately after admission. Women with eGDM had their follow-up visits scheduled every two to three weeks in the outpatients’ clinic. If glycemic targets were not met at the first follow-up visit, insulin therapy in a basal-bolus mode was added to the diet.

We retrieved data from the hospital records regarding maternal history and anthropometrics: prepregnancy body weight, height, and glycemic measurements during the 75 g OGTT (transferred to the documentation from maternal records at the first admission to the Unit). All patients who met the IADPSG criteria for GDM (at least one of the following: fasting glycemia ≥ 5.1 mmol/dL, 1-h plasma glucose in 75 g OGTT ≥ 10 mmol/dL, 2-h plasma glucose in 75 g OGTT ≥ 8.5 mmol/dL) were diagnosed with eGDM [23]. For a negligible proportion of the cohort referred to the unit before 2010, we used the contemporary criteria used locally in Poland (fasting glycemia ≥ 5.3 mmol/dL, or 2-h glycemia in 75 g OGG ≥ 7.8 mmol/dL).

According to the IADPSG recommendations, patients who presented glucose levels diagnostic of overt diabetes according to the WHO 2013 criteria (i.e., fasting glycemia of 7 mmol/dL or more, or 2-h glycemia in 75 g OGTT, above 11.1 mmol/dL), were defined as the eDiP arm of the study [23].

Maternal body weight at term was retrieved from the documentation regarding delivery. Gestational weight gain (GWG) was measured by subtracting the bodyweight recorded in a patient’s pregnancy chart (a mother’s pass issued by an obstetrician providing routine antenatal care for a patient) at the first antenatal visit (usually before a gestational age of 8 weeks) from the actual body weight measured at the last antenatal visit before the delivery. In accordance with the Institute of Medicine, we defined excessive gestational body weight gain as exceeding prepregnancy BMI-related GWG, according to the Institute of Medicine [24]. Restricted GWG was defined as GWG below recommendations of the IOM adjusted for the prepregnancy maternal BMI [24].

We also referred GWG in our cohort to the more recent observations regarding optimal GWG across BMI categories provided by the LifeCycle Project [25]. Thus, we created the “GWGLCP excessive/optimal/restricted” categories if maternal GWG exceeded, falling within the limits set by the LifeCycle Project, or was below the limits found by the study for a particular BMI category, respectively.

Lipid levels and 10-point daily glycemic profiles were measured during the first appointment to the Department (baseline maternal metabolic characteristics). From the lipid levels recorded during hospitalizations before a gestational age of 20 weeks, we calculated the following lipid ratios: Dobiasova index (also known as the atherogenic index of plasma–AIP, calculated as Log(triglycerides/HDL cholesterol)), total/HDL cholesterol ratio, non-HDL/HDL cholesterol ratio, and triglycerides/HDL cholesterol ratio [26,27,28]. We considered the lipid profile as unfavorable if at least one of the following occurred: AIP > 0.24, nonHDL/HDL cholesterol > 2.6, TOTAL/HDL ratio > 4.5, triglycerides/HDL cholesterol ratio > 1.65 [21,26,29].

To estimate insulin-resistance in our cohort, we analyzed the prevalence of the following dysmetabolic traits considered to be surrogate markers of the condition: prepregnancy BMI ≥ 30 kg/m^2^, estimated glucose disposal rate at baseline (eGDR, calculated using the formula with prepregnancy BMI and HbA_1c_ measured during the first visit to the tertiary-level Unit) below 8.77, fasting glucose above 5.6 mmol/dL, serum triglycerides above 1.7 mmol/dL, HDL cholesterol below 1.3 mmol/dL, and blood pressure above 130/85 mmHg (or on antihypertensive medications) [30].

We diagnosed an adverse maternal outcome (AMO) if at least one of the following maternal end-points occurred: gestational hypertension (PIH), preeclampsia (PET), or medically-indicated prematurity (delivery before 37 completed weeks of gestation due to maternal indications).

We also retrieved data regarding newborns from the documentation regarding delivery. We diagnosed an adverse neonatal outcome (ANO) if at least one of the following neonatal end-points occurred: birth weight above the 90th percentile calculated according to the WHO international growth charts (LGA), birth weight below the 10th centile according to the WHO international growth charts (SGA), emergency caesarean section (eCS) for fetal indications, medically indicated prematurity (delivery before 37 completed weeks of gestation due to neonatal indications), neonatal hypoglycemia (neonatal glycemia measured in the first hours of life which prompted a decision about a treatment), or neonatal hyperbilirubinemia requiring phototherapy. Neonatal hypoglycemia was coded together with hyperbilirubinemia as an adverse neonatal metabolic outcome (mANO). Cases in which respiratory support was necessary were analyzed together as an adverse respiratory neonatal outcome (rANO). Cases in which any ANOs occurred were defined as a composite adverse neonatal outcome (cANO). We calculated a Ponderal Index (PI) from neonatal anthropometrics, and considered a PI above 3.0 g/cm^3^ as a high PI, and a PI below 2.2 g/cm^3^ as a low PI [31,32]. To refer birth weight in our cohort to the general population, we calculated z-scores for birth weights. A Z-score above 2.0 was defined as a high z-score, and a z-score below −2.0 was defined as a low z-score [33].

Statistical analysis was performed with SPSS 18.0 for Windows (SPSS Inc., Chicago, IL, USA). Data were checked for normality using the D’Agostino–Pearson test. All variables, except baseline diastolic blood pressure, gestational age at the diagnosis of hyperglycemia, maternal age, maternal height, baseline AIP, and baseline HDL cholesterol, were not normally distributed. The odds ratio and 95% confidence intervals were calculated for proportions of maternal complications. A univariate analysis and ROC curve analysis were carried out as screening tests to identify variables related to the outcomes initially. Next, multivariate logistic regression models using all variables related to the outcomes with a *p* < 0.1 were used to identify predictors for adverse maternal or neonatal outcomes coded dichotomously. Variables are presented as mean ± standard deviation or median (interquartile range). *p* < 0.05 was considered statistically significant, and the Benjamini–Hochberg procedure was performed to control for multiplicity, with a false discovery rate of 5%.

In a post-hoc study power calculation, we confirmed that our population size enabled a sufficient power (>90%) to detect a difference in hypertensive disorders of pregnancy, emergency cesarean section, and NICU hospitalization of the newborn, compared to the prevalence of these complications confirmed in a recent study of a large cohort of lean, normoglycemic pregnant women [34].

## 3. Results

Characteristics of the study group are summarized in Table 1.

Our cohort was predominantly overweight or obese. None of our patients reported smoking in pregnancy, or being on hypolipemic medications before pregnancy.

In 21% of women, we diagnosed diabetes in pregnancy (eDiP), according to the WHO/IADPSG criteria. Among the patients, 60.8% required insulin therapy at enrolment, or added to the diet throughout pregnancy. Comparing baseline characteristics between eGDM and eDiP subgroups, we found a significant difference in the following biomarkers of insulin resistance: early pregnancy eGDR (9.33 ± 1.56 vs. 7.92 ± 2.54, respectively, *p* = 0.007) and in a proportion of patients with eGDR < 8.77 (28.6% vs. 55.2%, respectively, *p* = 0.014). Also, the TAG/HDL ratio was significantly lower in the eGDM arm compared to the eDiP subgroup (1.06 ± 0.78, vs. 1.25 ± 0.68, respectively, *p* = 0.020). A significant difference between the groups was also noted for AIP (−0.06 ± 0.25 vs. 0.04 ± 0.23 *p* = 0.021). Multiple dysmetabolic traits were more prevalent in patients with eDiP: three or more components of metabolic syndrome were present in 13.0% of patients with eGDM compared to 38.7% of those with eDiP, *p* = 0.007. Neither subgroup differed in terms of the prevalence of arterial hypertension (11.6% for eGDM vs. 9.8% for eDiP), gestational hypertension/preeclampsia (7.5% vs. 7.3%, respectively), prepregnancy BMI of 30 kg/m^2^_,_ or more (40.8% vs. 44.7%, respectively), and maternal history of GDM in previous pregnancies (20.5% vs. 29.2%, respectively).

Gestational hypertension or preeclampsia was found in 7.7% of the cohort. Comparing maternal characteristics between women who developed gestational hypertension, either/or preeclampsia, and those who did not, we did not notice any significant differences in baseline maternal anthropometrics and glycemic control. Moreover, surrogate markers of insulin resistance measured in the first trimester did not differ between these groups.

Excessive GWG occurred in 17.2% of the participants for which data on prepregnancy and late pregnancy body weight were available (N = 98) if calculated according to the IOM criteria, and in 22.2% of these patients if calculated according to the data from the LCP.

Comparing baseline maternal characteristics, we did not find any differences between the subgroup with a gestational weight gain exceeding the recommendations of the IOM, and the patients with the GWG within, or below, the recommendations, apart from the statistical difference in the gestational age at the diagnosis of GDM: 12.0 gestational weeks (10.0; 15.0) for patients with GWG within or below the recommendations, compared to 10.0 gestational weeks (6.0; 12.0) in women with excessive GWG (*p* = 0.013).

Restricted GWG according to the IOM criteria was found in 51 out of 98 (54.3%) patients, and 30.3% patients if the LCP criteria were used. Comparing maternal characteristics between women with restricted gestational weight gain and women with appropriate or excessive weight gain, we did not notice any significant differences in baseline maternal anthropometrics and glycemic control. Moreover, surrogate markers of insulin resistance measured in the first trimester did not differ between these groups.

Descriptive analysis of the neonatal outcomes in our cohort (Table 2) confirms that the population we have studied is a high-risk population for neonatal complications: at least one adverse outcome occurred in six out of ten neonates. This maternal population is also a source of medically-indicated prematurity: less than half of premature deliveries occurred spontaneously.

Metabolic adverse neonatal outcome (mANO) was diagnosed in 41.4% of the newborns. Comparing maternal baseline characteristics between the subgroup with and without this adverse outcome, we found significant differences in several lipids and lipid ratios. The women who gave birth to newborns with mANO had significantly higher serum levels of total cholesterol (5.3 (4.6; 6.2) mmol/dL vs. 4.8 (4.1; 5.5) mmol/dL *p* = 0.044), significantly higher serum levels of triglycerides (1.8 (1.3; 2.8) mmol/dL vs. 1.3 (1.0; 1.7) mmol/dL *p* = 0.003), significantly higher serum levels of non-HDL cholesterol (3.6 (2.9; 4.4) mmol/dL vs. 2.9 (2.6; 3.8) mmol/dL *p* = 0.015), significantly higher triglycerides/HDL-cholesterol ratio (1.0 (0.7; 1.9) vs. 0.7 (0.5; 1.0) *p* = 0.009), significantly higher non-HDL/HDL cholesterol ratio (2.4 (1.7; 3.0) vs. 1.9 (1.6; 2.1) *p* = 0.019), significantly higher total/HDL cholesterol ratio (3.4 (2.7; 4.0) vs. 2.9 (2.6; 3.1) *p* = 0.019), and significantly higher AIP (0.00 (−0.13; 0.27) vs. −0.13 (−0.28; 0.02) *p* = 0.008).

In the analysis of the ROC curves, all these parameters significantly, albeit weakly, predicted the outcome (AUC for TAG/HDL cholesterol ratio: 0.66, *p* = 0.013, AUC for non-HDL/HDL cholesterol ratio: 0.65, *p* = 0.019, AUC for total/HDL cholesterol ratio: 0.65, *p* = 0.019, AUC for AIP: 0.66, *p* = 0.012). However, in the multivariate analysis, only a non-HDL/HDL cholesterol ratio measured at the baseline significantly predicted mANO (OR: 2.3 (1.2; 4.5), *p* = 0.012, *p* for the model= 0.006, Nagelkerks’s R^2^ = 0.122).

Emergency caesarean section (eCS) for neonatal reasons occurred in 16.9% of the caesarean deliveries. Women who required such eCS had significantly higher 2-h glucose levels in 75 g OGTT (192.0 (166.0; 234.5) mg/dL vs. 157.0 (115.5; 183.0) mg/dL, *p* = 0.033), and a significantly lower prepregnancy BMI (24.6 (22.8; 29.4) kg/m^2^ vs. 30.8 (26.3; 34.8) kg/m^2^, *p* = 0.025). The analysis of the ROC curves revealed that both parameters significantly predicted the outcome (AUC for BMI: 0.15, *p* = 0.012, AUC for 2-h glucose level in the 75 g OGTT: 0.79, *p* = 0.036). In the multivariate analysis, both parameters predicted this outcome with a borderline significance (OR for the glucose levels: 1.10 (1.00; 1.22) mg/dL *p* = 0.058, OR for BMI: 0.39 (0.15; 1.01) *p* = 0.052), but the model explained a large proportion of the variance of the dependent variable (*p* for the model < 0.001, R^2^ = 0.72).

A high z-score for a birth weight was diagnosed in 9.5% of newborns. Women who gave birth to the neonates with a z-score above 2.0 had significantly lower baseline eGDR (7.32 (5.28; 9.50) vs. 9.33 (7.91; 10.45), *p* = 0.012), significantly higher baseline HbA1c (6.2 (5.2; 7.9)% vs. 5.5 (5.1; 5.9)% *p* = 0.049, non-significant after performing the Benjamini–Hochberg procedure), and hyperglycemia was diagnosed significantly earlier in this subgroup (10.0 (6.0; 12.0) vs. 12.0 (10.0; 15.0), *p* = 0.035). We also noted a trend towards a higher maternal prepregnancy BMI in the subgroup of neonates with a z-score above 2.0 (31.4 (27.2; 37.5) kg/m^2^ vs. 28.1 (23.7; 32.3) kg/m^2^, *p* = 0.066). In the analysis of the ROC curves, we confirmed that eGDR, gestational age at the diagnosis, and HbA1c significantly predicted the outcome (AUC for eGDR: 0.28, *p* = 0.012; AUC for the gestational age: 0.33, *p* = 0.036; AUC for HbA1c: 0.67, *p* = 0.049). In the multivariate analysis, both baseline eGDR and gestational age at the diagnosis of hyperglycemia remained statistically significant, negative predictors for a high z-score (OR for the eGDR: 0.72 (0.55; 0.93), *p* = 0.011; OR for the gestational age at the diagnosis: 0.82 (0.68; 0.99), *p* = 0.039). However, this model explained only 16.5% of the variance in the dependent variable.

A low ponderal index (PI) was diagnosed in 76.2% of newborns, and none of the neonates had a high PI. None of the maternal parameters correlated with the risk of a low PI.

Table 3 presents the results of a univariate analysis regarding dysmetabolic traits and markers of an unfavorable lipid profile as predictors for an adverse neonatal outcome. The analysis indicated that several dysmetabolic traits in early pregnancy were linked to a significantly increased risk of neonatal complications. However, these maternal predictors explained only a small proportion of the variance of the dependent variables, and no longer remained statistically significant in the multivariate analysis. Only a 2-h glycemia in 75 g OGTT strongly correlated with the risk of emergency CS for neonatal indications.

## 4. Discussion

Our study presents observational data from a cohort of pregnant women with hyperglycemia diagnosed in early pregnancy. The major strength of our study is that it provides comprehensive descriptive data regarding the metabolic profile of early pregnancy and its association with fetomaternal outcome in a specific cohort of pregnant women, which is not widely studied.

Our observations confirm that several metabolic traits suggestive of insulin resistance were widely prevalent in this specific cohort: obesity, hypertension, fasting glycemia > 5.5 mmol/dL, and reduced glucose uptake in the peripheral tissues. Our patients have shown multiple metabolic traits typical for an atherogenic lipid profile, which cannot be explained solely due to gestational adaptation driven by placental hormones. The measurements were performed in early pregnancy before the placenta fully develops its endocrine function [35].

Importantly, we confirmed that women with eDiP differ from those with eGDM regarding insulin resistance and atherogenicity. Overt hyperglycemia (eDiP) was associated with more severe insulin resistance, and a higher prevalence of atherogenic traits in women of an age and BMI similar to those noted in patients with mild hyperglycemia (eGDM). This observation suggests that women with eDiP present an early phenotype of type 2 diabetes rather than type 1, or MODY, which remains in line with data presented by other authors [36,37,38]. Interestingly, although women with eDiP differed from those with eGDM with respect to insulin resistance and glucose metabolism, both subgroups had a similar proportion of women with chronic and gestational hypertension. This observation suggests that timely treatment of hyperglycemia and stabilization of gestational weight gain could contribute to improved perinatal outcomes in the subgroup with eDiP. Also, it should be stated that although the arm of eDiP presented a more severe phenotype of insulin resistance, eGDM could not be considered as a purely biochemical finding. This subgroup also presents a prevalence of metabolic risk factors above those expected in a population of young women. Moreover, one needs to be aware that maternal hypeglycemia is a continuous risk factor for fetal and neonatal complications: all thresholds set for the diagnosis of maternal hyperglycemia are arbitrary ones. Therefore, metabolic risk factors are also likely to be present in women without diagnosis, possibly explaining some proportion of fetomaternal complications in women diagnosed as normoglycemic in pregnancy.

Our data also indicate that maternal glycemic status and lipid profile at baseline shaped the risks for adverse neonatal outcomes in the study group. Importantly, 2-h glycemia from the 75 g OGTT significantly predicted emergency cesarean section for neonatal indications, and was the only predictor for the composite neonatal adverse outcome. Interestingly, we noted that the atherogenic lipid profile at the baseline was linked to an increased risk of mild metabolic neonatal complications, such as hypoglycemia either/or hyperbilirubinemia. However, these associations failed to reach significance in a multivariate analysis; they explained only a small proportion of the variation in the outcome, and wide confidence intervals indicate that a point estimate was measured in a small study population. Therefore, our finding regarding the lipid ratios and neonatal outcome in eGDM/eDiP population should be considered rather as a hypothesis-generating study.

Our cohort of patients with hyperglycemia diagnosed in early pregnancy differs from populations with hyperglycemia diagnosed in the third trimester, as regards an unusually high proportion of newborns with a ponderal index suggestive of SGA [31]. Studies of patients with GDM describe a pattern of a predominant high PI, suggestive of fetal adiposity [10]. However, none of the parameters we investigated in our cohort correlated with a low PI. Thus, our observation suggests that this variable might not be regulated epigenetically. Nevertheless, adequately powered studies performed in larger cohorts similar to ours in terms of early diagnosed gestational hyperglycemia could be necessary to corroborate our observation.

Our study also delivers early data on lipid ratios and their impact on maternal or neonatal outcomes. Such an approach is rare in studies regarding pregnancy, where available evidence linked lipid levels in pregnancy to an elevated risk for preterm birth mainly analyzed lipid levels [39,40,41]. Moreover, observations from the ROLO study, which confirmed a positive association between maternal triglycerides and birth weight, analyzed raw lipid levels [14]. Lipid ratios are widely studied outside pregnancy, and are well-documented risks for increased cardiovascular mortality and morbidity [29]. Although pregnancy constitutes a specific hormonal milieu, with hyperlipidemia as a physiological phenomenon, our data confirm that thresholds used outside pregnancy as indicators of the atherogenic lipid profile are also useful predictors of adverse neonatal outcomes in the high-risk population of pregnant women [35]. Hence, our data define possible areas for future interventions to reduce the lipid-related risk of adverse neonatal outcomes.

Although insulin resistance is a natural feature of a normal pregnancy, our study confirms that general population-based surrogate markers of the condition can be helpful as predictors for perinatal risk in this particular cohort if measured in early pregnancy. Similar observations were made by Franzago et al., who reported an association between the third-trimester triglycerides and carotid intima-media thickness after a three-year follow-up [40].

Our study has some limitations, mainly resulting from its retrospective design, such as a lack of measured insulin levels, which is not routinely performed in pregnant women. We are aware that an investigation of insulin resistance calculated directly from the glucose and insulin concentrations would probably provide more accurate results. However, the lipid rations we used were confirmed as reliable proxies for the condition in the nonpregnant female population [22,42]. eGDR was investigated as a reliable proxy for insulin resistance in patients with type 2 diabetes [43,44]. However, we had to use the alternative formula, not used in pregnant women, to avoid the measurement of waist circumference, which can be misleading even in early pregnancy [30]. Another limitation comes from the small size of our cohort, which means that these observations need to be replicated in larger, adequately powered cohorts, using recommended thresholds for several neonatal outcomes, such as hypoglycemia. However, the body of evidence investigating the metabolic syndrome in early pregnancy is limited. Therefore, we believe that these data can still offer a useful starting point for future studies, or contribute to meta-analyses.

Apart from these limitations, the results from our cohort confirm that maternal hyperglycemia in early pregnancy is not an isolated clinical symptom. Our data show that it coexists with complex metabolic derangement regarding the lipid profile, particularly elevated triglycerides or an elevated nonHDL/HDL cholesterol ratio. Moreover, one-fifth of our patients presented an early-pregnancy HbA1c above 6%, which has been identified as a risk factor for cardiovascular mortality in the non-diabetic population [45]. Hence, our observations support a need for taking pregnancy as an opportunity to improve long-term maternal and neonatal health [5].

## Figures and Tables

**Table 1:** Maternal characteristics of the study group; data presented as median (interquartile range), or %.

Parameter	N = 193
Age (years)	31 (28.0; 34.0)
Prepregnancy BMI (kg/m^2^)	29.2 (24.1; 33.6)
GDM in previous pregnancy/pregnancies (% out of women with a history of minimum one delivery)	22.5%
Gestational hypertension/preeclampsia diagnosed in this pregnancy	7.7%
Multipara (%)	56.4%
History of T2DM in the family	45.1%
Gestational age at the diagnosis of hyperglycemia in pregnancy (weeks)	12.0 (9.0; 15.0)
Fasting glycemia at the diagnosis (mmol/dL)	5.6 (5.2; 6.3)
1-h glycemia in the 75 g OGTT (mmol/dL)	9.3 (8.2; 10.7)
2-h glycemia in the 75 g OGTT (mmol/dL)	9.0 (7.1; 10.5)
Maternal metabolic characteristics at the baseline (the first visit in the tertiary Unit completed before the gestational age of 20 gestational weeks)	
HDL cholesterol (mmol/dL)	1.7 (1.4; 2.1)
Total cholesterol (mmol/dL)	5.1 (4.4; 5.7)
Triglycerides (mmol/dL)	1.5 (1.1; 2.0)
HbA1c (%)	5.4 (5.1; 5.9)
TAG/HDL cholesterol ratio	0.84 (0.64; 1.30)
nonHDL cholesterol/HDL cholesterol ratio	2.00 (1.62; 2.50)
Total cholesterol/HDL cholesterol ratio	3.00 (2.62; 3.50)
Atherogenic Plasma Index	−0.07 (10.19; 0.11)
eGDR	9.12 (7.48; 0.38)
Blood pressure > 130/85, or on antihypertensive medications	14.6%
Fasting blood glycemia > 5.5 mmol/dL	53.2%
HDL cholesterol < 1.29 mmol/dL	16.4%
TAG > 1.69 mmol/Dl	47.5%
Prepregnancy BMI > 30 kg/m^2^	44.9%
eGDR < 8.77	39.3%
TAG/HDL cholesterol ratio > 1.65	15.4%
AIP > 0.24	14.6%
nonHDL/HDL cholesterol ratio > 2.6	23.5%
TOTAL/HDL cholesterol ratio > 4.5	9.2%

List of abbreviations: BMI, body mass index; eGDR, estimated glucose disposal rate; GDM, gestational diabetes; HbA1c, glycated hemoglobin; OGTT, oral glucose tolerance test; T2DM, type 2 diabetes mellitus; TAG, triglycerides.

**Table 2:** Neonatal characteristics of the study group; data presented as median (interquartile range), or %.

Parameter	
Neonatal sex	Female/male 44.1/55.9%
Birth weight (g)	3450 (3110; 3757)
Z-score for a birth weight	0.48 (−0.19; 1.13)
Z-score below −2.0	0%
Z-score above 2.0	9.6%
Ponderall Index (cm/g^3^)	2.06 (1.88; 2.20)
Low Ponderall Index (2.2 g/cm^3^, or less)	76.2%
High Ponderall Index (3.0 g/cm^3^, or more)	0%
Gestational age at delivery (gestational weeks)	38.5 (38.0; 39.0)
Birth weight > 90th centile according to the WHO growth charts (%)	25.2%
Birth weight < 10th centile according to the WHO growth charts (%)	9.2%
Emergency caesarean section (CS) for fetal indications (out of CSs) (%)	16.9%
Metabolic adverse neonatal outcome (neonatal hypoglycemia either/or hyperbilirubinemia/phototherapy) (%)	41.4%
Postpartum birth weight loss above 10% (%)	9.4%
Intrapartum injury (%)	13.0%
Premature delivery < 37 weeks of gestation (%)	9.2%
Premature delivery < 34 weeks of gestation (%)	4.5%
Medically-indicated prematurity (delivery below 37 weeks of gestation for fetal indications) out of premature deliveries (%)	54.5%
Respiratory adverse neonatal outcome (respiratory support (CPAP), either/or mechanical ventilation) (%)	6.2%
Composite adverse neonatal outcome (%)	59.2%

**Table 3:** Maternal characteristics and markers of insulin resistance as predictors of the neonatal outcomes in the study group; data from the univariate logistic regression.

Neonatal Outcome	Predictor	Exp(B) (95% CI)	Nagelkerke R^2^	*p*
Adverse metabolic neonatal outcome	75 g OGTT glycemia at 2 h diagnostic for DiP *p for the model = 0.006*	1.59 (1.48; 16.59)	0.102	*0.011*
	nonHDL/HDL cholesterol > 2.6 *p for the model = 0.009*	4.62 (1.35; 15.79)	0.110	*0.014*
	AIP > 0.24 *p for the model = 0.033*	3.60 (1.04; 12.48)	0.072	*0.043*
	TOTAL/HDL cholesterol ratio > 4.5 *p for the mode = 0.015*	8.75 (1.02; 74.83)	0.095	*0.048*
	Three or more dysmetabolic traits present at the baseline *p for the model = 0.024*	3.91 (1.12; 13.65)	0.085	*0.032*
Composite adverse neonatal outcome *p for the model = 0.010*	75 g OGTT glycemia at 2 h diagnostic for DiP	4.56 (1.24; 16.74)	0.080	*0.022*
High z-score	Gestational age at the diagnosis of hyperglycemia < 12 weeks *p for the model = 0.021*	1.58 (0.79; 4.03)	0.08	*0.045*
	Baseline HbA1c > 6.5% *p for the model = 0.052*	4.07 (1.08; 15.28)	0.060	*0.037*
	Baseline Egdr < 8.77 *p for the model = 0.025*	4.36 (1.10; 17.32)	0.089	*0.036*

List of abbreviations: AIP, atherogenic index of plasma; DiP, diabetes diagnosed in pregnancy; eGDR, estimated glucose disposal rate; HbA1c, glycated hemoglobin; OGTT, oral glucose tolerance test; all *p*-values remained significant after performing the Benjamini–Hochberg procedure.

## Data Availability

The data presented in this study are available on request from the last author.
